# Sequel to the Case of Habitual Constipation

**Published:** 1876-05

**Authors:** Thomas D. Strong

**Affiliations:** Westfield, N.Y.


Sequel to the Case of Habitual Constipation Reported in the American Journal of the Medical Sciences for October, 1874.—*By Thomas D. Strong, M.D., of Westfield, N. Y.*—Milton Brooks, aged twenty-eight, died at Irving, Chautauqua county, New York, January 21st, 1876.

The last report of the case brought down the history of it to June 1, 1874. Since that time the frequency of dejections has increased, so that his habit has been from five to ten days, though he has gone a month or more once or twice.

Has had a good appetite most of the time, and for the past year has done more work than in any year before. A year ago he worked a team in railroad construction. He has increased in weight by adipose deposit. His last sickness was eight days, the attack being like those in former report, violent pain in stomach and bowels, vomiting, and after a short time diarrhœa.

Post-mortem was made seventeen hours after death, by Drs. Rogers, of Dunkirk, Thompson, of Angola, and myself.

Rigor mortis well defined. Most marked appearance of body was its breadth at base of chest, the ribs being widely spread; in fact, almost horizontal. The breadth at base of ribs was two inches greater than at superior spinous processes. The walls of abdomen had one inch of fat, and there was also a heavy deposit upon intestines, especially upon mesocolon. On opening chest and abdomen, we found that the arch of the diaphragm extended high into the chest, one inch above level of the nipple. Lungs compressed into the upper and back part of the thorax, looking like lungs compressed by heavy pleuritic effusion. Heart healthy. Left kidney deformed by pressure, shaped like section of a pyramid; the right elevated three inches above natural position, both healthy in structure. Liver one-half larger than normal, otherwise appearing healthy. Spleen somewhat enlarged. Stomach healthy in size and texture.

Although he had diarrhœa, with profuse evacuations, during most of the eight days of his sickness, we emptied from the colon, after removing it, six quarts of semifluid fecal matter. The colon measured, undistended, six feet three inches in length, and thirteen inches in circumference. Two feet of the lower extremity were much thickened. The mucous membrane of the whole colon was intensely congested.

During the last two or three years of *partial* distension, there can have been but little return by the organs to their normal position or size.—*Amer. Jour. Med. Sciences.*

